# Sensitivity and specificity of malaria rapid diagnostic test (mRDT CareStat^TM^) compared with microscopy amongst under five children attending a primary care clinic in southern Nigeria

**DOI:** 10.4102/phcfm.v12i1.2212

**Published:** 2020-06-17

**Authors:** Oluwagbenga Ogunfowokan, Bamidele A. Ogunfowokan, Anthony I. Nwajei

**Affiliations:** 1 Lincoln University College, Selangor, Malaysia; 2 The Ark Medical Centre, Asaba, Nigeria; 3 Federal Medical Centre, Asaba, Nigeria

**Keywords:** family medicine, primary care, education, mRDT, parasite density, sensitivity, specificity

## Abstract

**Background:**

Malaria diagnosis using microscopy is currently the gold standard. However, malaria rapid diagnostic tests (mRDTs) were developed to simplify the diagnosis in regions without access to functional microscopy.

**Aim:**

The objective of this study was to compare the diagnostic accuracy of mRDT CareStat^TM^ with microscopy.

**Setting:**

This study was conducted in the paediatric primary care clinic of the Federal Medical Centre, Asaba, Nigeria.

**Methods:**

A cross-sectional study for diagnostic accuracy was conducted from May 2016 to October 2016. Ninety-eight participants were involved to obtain a precision of 5%, sensitivity of mRDT CareStat^TM^ of 95% from published work and 95% level of confidence after adjusting for 20% non-response rate or missing data. Consecutive participants were tested using both microscopy and mRDT. The results were analysed using EPI Info Version 7.

**Results:**

A total of 98 children aged 3–59 months were enrolled. Malaria prevalence was found to be 53% (95% confidence interval [CI] = 46% – 60%), whilst sensitivity and specificity were 29% (95% CI = 20% – 38%) and 89% (95% CI = 83% – 95%), respectively. The positive and negative predictive values were 75% (95% CI = 66.4% – 83.6%) and 53% (95% CI = 46% – 60%), respectively.

**Conclusion:**

Agreement between malaria parasitaemia using microscopy and mRDT positivity increased with increase in the parasite density. The mRDT might be negative when malaria parasite density using microscopy is low.

## Introduction

### Background

Malaria is one of the leading causes of morbidity and mortality worldwide, especially in under 5-year-old children and pregnant women in sub-Saharan Africa, where over 80% of cases and at least 90% of malaria deaths occur.^1,2^ The basic elements of malaria case control or management include early diagnosis and prompt treatment. The gold standard for malaria diagnosis has been light microscopy examination of Giemsa-stained blood smear for malaria parasites. Microscopy detects the actual parasite and the different species of the plasmodium. However, because of lack of equipment (microscopes and power source) and trained microscopists in most malaria endemic regions, malaria rapid diagnostic tests (mRDTs) were developed to fill this gap. The malaria rapid diagnostic test is an immunochromatographic test relying principally on the capture of the target malaria antigen from the blood specimen of the patient.

### Objectives

The objectives of this study were to determine the sensitivity and specificity of mRDT and factors that affect these parameters using microscopy as the gold standard, to calculate the positive and negative predictive values of mRDT in the study population and to determine the association between parasite density and mRDT.

### Hypothesis

We hypothesized that mRDT has sensitivity and specificity similar to microscopy.

### Setting

The study was conducted in the Children Outpatient Clinic (CHOP) of the Federal Medical Centre, Asaba. The clinic has three consulting rooms that are run by family physicians (Residents and Consultants) in the Family Medicine department. An average of 100 malaria cases are seen monthly amongst under five children presenting to the clinic. Asaba is the capital city of Delta State and shares boundary with Anambra State on the eastern coast of the Niger River.

### Rationale

Although some work has been carried out on this topic, there is paucity of published work on the subject in the South – South geopolitical zone of Nigeria, and the adoption of the mRDT is yet to be popular. This study is therefore aimed at bridging this gap and also to find out the parasite density at which the mRDT will become positive with the aim of making recommendations for its adoption during diagnosis of malaria, especially where the microscopic diagnosis is not feasible.

## Research methods and design

### Study design

The study was a cross-sectional study, comparing diagnostic accuracy of malaria using mRDT CareStat^TM^ (a histidine-rich protein-2 *Plasmodium falciparum*-based kit) and microscopy conducted in the Children Outpatient Clinic (CHOP) of the Federal Medical Centre, Asaba. The kit was preferred as the *falciparum* species is the predominant species in the west African subregion. Ethical clearance was obtained from the ethical committee of the Federal Medical Centre Asaba (FMC/ASB/T/A81/66).

Participants were consecutively recruited into the study until the desired sample size was achieved. Finger prick blood sample was obtained, and one thin and one thick blood smears were prepared, stained with 10% Giemsa and read for the presence of malaria parasite by a microscopist. The microscopist was a qualified laboratory scientist with the Federal Medical Centre, Asaba. He has been doing malaria microscopy for 10 years and has been certified as a malaria microscopist. Ten out of the 98 slides were also randomly selected and sent to the parasitology unit of the Medical Microbiology Laboratory of the University College Hospital Ibadan. A drop of blood (about 5 µL) was also taken from the thumb by a dropper that came with the rapid diagnostic test kit. The malaria rapid tests were conducted by the second author, strictly following the instructions on the leaflet in the CareStart^TM^ packs. The sample was introduced into the kit chamber and two drops of the buffer solution were introduced and left for 5–10 min. The results were read and recorded as positive or negative for malaria parasite. The microscopist was independent and hence not aware of the mRDT results obtained. This was performed to remove bias in his interpretation of the blood films sent to the laboratory. The results of the microscopy and the mRDT tests were compared using EPI Info^TM^ 7 (7.1.5) and the data were summarised using proportions, frequency and percentages. The sensitivity, specificity and predictive values of mRDT were compared with that of the microscopy. Regression analysis was used to determine the relationship between malaria parasite density and the positivity of mRDT.

### Setting

The CHOP of the Federal Medical Centre, Asaba, Delta State, Nigeria, is being run by the Family Medicine Department, and it provides primary care delivery for children attending the hospital. The hospital is situated along Nnebisi road in the west-end area of the town close to Saint Patrick College, Asaba.

### Study population

The study population were children who were under 5 years of age attending the Children Outpatient Clinic of the Federal Medical Centre, Asaba. The inclusion criteria were as follows: children whose parents or guardian provided informed consent, children aged between 3 and 59 months, children with axillary temperature ≥ 37.5 °C at presentation or a history of fever within the previous 48 h, children presenting with symptoms and signs comparable with the clinical picture of malaria. The exclusion criteria were as follows: refusal of the parents or guardians to provide informed consent, children with signs of severe illness or unconscious at presentation and those who were enrolled in other clinical studies.

### Data collection

The data were collected using an interviewer-administered questionnaire in English and a translation into pidgin English for parents who did not understand English.

### Data analysis

The data collected were analysed using the EPI Info Version 7 statistical package. Parasite density was assessed with the thick film, whilst parasite speciation was assessed with the thin film. The slide was considered negative when no parasite was seen or detected after screening 200 high power fields (see Table 1). Asexual stages of the malaria parasite on thick films were counted against about 200 White Blood Cells (WBCs).^3^ The parasite density (parasites/µL of blood) was calculated according to the formula below using the World health organization (WHO) recommended assumed WBC count of 8000/µL of blood.

$$\begin{array}{lll} \text{Definitive count} & = & \frac{\text{No. of asexual parasites counted}}{\text{No. of WBC counted}}\times 8000/\mu \text{L}\\ \; & = & \text{parasite density}/\mu \text{L} \end{array}$$ [Eqn 1]

**TABLE 1:** Diagnostic accuracy of the malaria rapid diagnostic test compared with microscopy.

mRDT Result (Index Test Result)	Microscopy Result (Reference Standard)		Total
	Positive	Negative	
Positive	(TP)	(FP)	(TP + FP)
Negative	(FN)	(TN)	(FN + TN)
**Total**	**TP + FN**	**FP + TN**	**TP + FN + FP + TN**

FN, false negative; FP, false positive; mRDT, malaria rapid diagnostic test; TN, true negative; TP, true positive.

Sensitivity = TP / (TP + FN) X 100.

Specificity = TN / (FP + TN) X 100.

Positive predictive value = TP / (TP + FP) X 100.

Negative predictive value = TN / (FN + TN).

### Ethical consideration

Ethical clearance to conduct the study was obtained from the Federal Medical Centre, Asaba, Nigeria (Ethical Clearance Number: FMC/ASB/T/A81/66) on 16 December 2015. The study was conducted in compliance with International Conference on Harmonisation – Good Clinical Practice (ICH GCP) and the Declaration of Helsinki.

## Results

A total of 98 children aged between 3 and 59 months were recruited and enrolled in this study. There were 53 males and 45 females, giving a male–female ratio of 1:0.85. Most of the participants were in the age group of 3–24 months, accounting for 50 members (51.02%) of the total participants. The mean age ± standard deviation (s.d.) of the participants was 26.2 months ± 15.7 (range = 3–59 months) as shown in Table 2.

**TABLE 2:** Age category of participants in months.

Age range(months)	Proportion	Percentage	Gender		Mean age	±s.d.
			Male	Female		
3–12	25	25.5	-	-	-	-
13–24	25	25.5	-	-	-	-
25–36	20	20.4	54	46	26.2	15.7
37–48	19	19.4	-	-	-	-
49–59	9	9.2	-	-	-	-
**Total**	**98**	**100**	**-**	**-**	**-**	**-**

s.d., standard deviation.

The most common clinical symptom was fever. Eighty-eight of the participants (89.8% [95% CI = 82.03% – 95.0%]) presented with fever. About 74.5% of the participants had raised temperature of ≥ 37.5 °C, with a mean ± s.d. temperature of 38.0 °C ± 0.9 °C (range = 36.0 °C – 39.9 °C) as shown in Table 3. *Plasmodium falciparum* was the only malaria species in this study, accounting for the entire positive malaria smear.

**TABLE 3:** Clinical features of the participants.

Characteristics	Frequency *N* = 98	Percentage	95% CI
**Symptoms**			
Fever			
At presentation	88	89.8	82.23–94.37
Before presentation	83	84.7	76.27–90.50
Refusal of feeds			
At presentation	65	66.3	56.52–74.91
Before presentation	51	52.0	42.26–61.67
Irritability			
At presentation	26	26.5	18.80–36.04
Before presentation	16	16.3	10.31–24.89
Vomiting			
At presentation	32	32.7	24.17–42.44
Before presentation	29	29.6	21.46–39.26
**Sign**			
Raised temperature > 37.5 °C			
At presentation	73	74.5	65.05–82.08
Mean temperature ±s.d.	38.0°C ± 0.9°C	-	36.0–39.9

s.d., standard deviation; CI, confidence interval.

**FIGURE 1:**
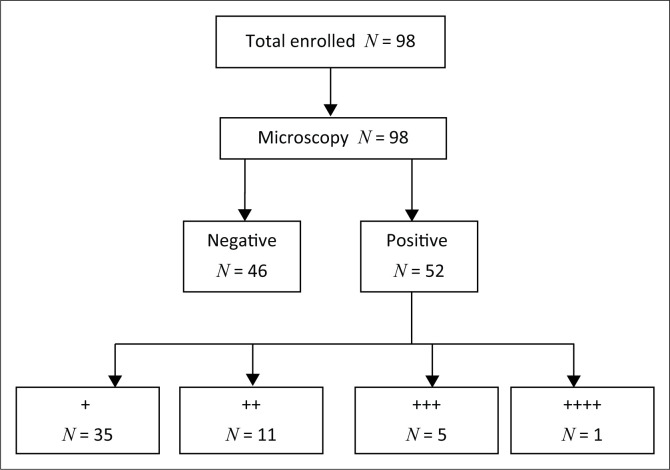
Flow chart showing the result of microscopy test (the gold standard).

**FIGURE 2:**
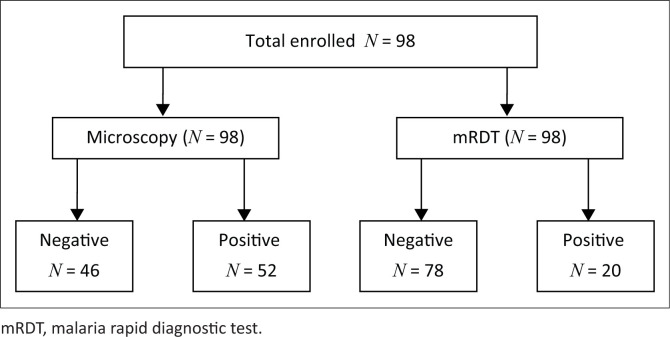
Comparison of the microscopy with the malaria rapid diagnostic test.

Both microscopy and mRDT were conducted on every participant. Fifty-two children (53.06% [95% CI = 40.72% – 61.26%]) out of 98 were found positive for the microscopy test, whilst 20 (20.41% [95% CI = 12.93% – 29.74%]) were positive with the mRDT test as shown in Figures 1 and 2.

Of the 20 participants who tested positive with the rapid diagnostic test, 19 gave a history of fever at the time of presentation. However, there was no significant relationship between fever and positivity of the rapid diagnostic test as shown in Table 4.

**TABLE 4:** Relationship between fever and malaria rapid diagnostic test positivity.

Fever at presentation	Positive mRDT				Total	Chi square	*p*
	Yes		No				
	*n*	%	*n*	%			
Yes	19	21.6	69	78.4	88	0.741	0.64
No	1	10%	9	90%	10	-	-
**Total**	**20**	**-**	**78**	**-**	**98**	**-**	**-**

mRDT, malaria rapid diagnostic test.

There was no significant relationship between the temperature at presentation and the malaria parasite count as shown in Table 5.

**TABLE 5:** Relationship between parasite count and temperature.

Temperature		Results of microscopy						Chi-square	*p*
		+	++	+++	++++	Nil	Total		
High temperature (≥ 37.5°C)		28	7	5	0.0	33	73	3.94	0.21
	%	38.4	9.6	6.8	0.0	45.2	100	-	-
Normal temperature (< 37.5°C)		7	4	0	1	13	25	-	-
	%	28.0	16.0	0.0	4.0	52.0	100	-	-
**Total**		**35**	**11**	**5**	**1**	**46**	**98**	**-**	**-**

**FIGURE 3:**
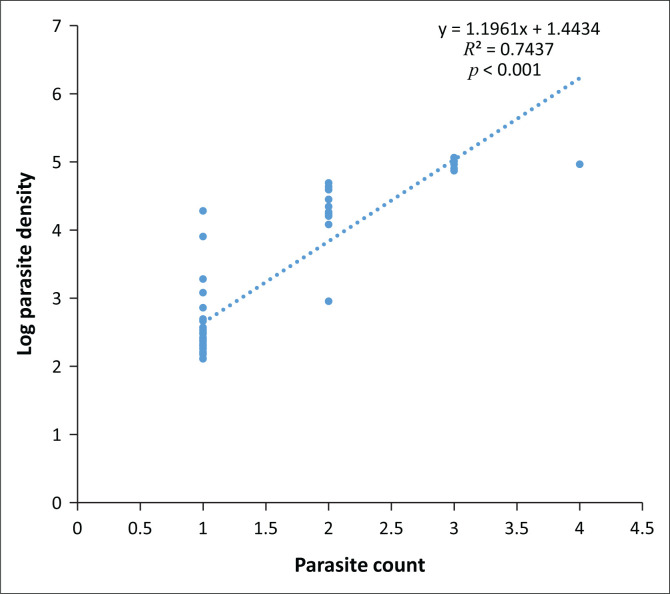
Linear regression of the parasite count against natural logarithm of parasite density of participants with malaria parasitaemia.

The linear regression plot of the relationship between the natural logarithm of the parasite density and the parasite count of the participants with malaria parasitaemia is shown in Figure 3. The log parasite density increased by a unit as the parasite count increased by 1.2 (*r*^2^ = 0.74, *p* < 0.001). The geometric mean parasite density of *P. falciparum* was 32 319 asexual parasites/µL.

The percentage agreement of positive results of mRDT and parasite count using microscopy of +, ++, +++ and ++++ was 14.3%, 45.5%, 80% and 100%, respectively. The percentage agreement of negative results was 10.9%. The proportion is shown in Table 6.

**TABLE 6:** Comparing malaria rapid diagnostic test positivity with parasite count.

Result mRDT	Results of microscopy										Total
	+		++		+++		++++		Nil		
	*n*	%	*n*	%	*n*	%	*n*	%	*n*	%	
Negative	30	85.3	6	54.6	1	20.0	0	-	41	89.4	78
Positive	5	14.3	5	45.5	4	80.0	1	100	5	10.9	20
**Total**	**35**	**-**	**11**	**-**	**5**	**-**	**1**	**-**	**46**	**-**	**98**

mRDT, malaria rapid diagnostic test.

The percentage agreement of positive results of mRDT and parasite count using microscopy is calculated as follows:

$$\frac{\text{No. of positive mRDT test}}{\text{Total positive test by microscopy}}\times 100$$ [Eqn 2]

- $$\text{mRDT}(+)=\frac{5}{35}\times 100=14.3\%$$ [Eqn 3]
- $$\text{mRDT}(++)=\frac{5}{11}\times 100=45.5\%$$ [Eqn 4]
- $$\text{mRDT}(+++)=\frac{4}{5}\times 100=80\%$$ [Eqn 5]
- $$\text{mRDT}(++++)=\frac{1}{1}\times 100=100\%$$ [Eqn 6]

The sensitivity and specificity of the mRDT were 29% and 89%, respectively, whilst the positive and negative predictive values were 75% and 54%, respectively. The prevalence of malaria using microscopy in this study population was 53%. The false positive and false negative rates were 10.9% and 71.2%, respectively, as shown in Table 7:

$$\begin{array}{lll} \text{Sensitivity} & = & \frac{TP}{TP+FN}\times 100\\ \; & = & \frac{15}{52}\times 100\\ \; & = & 28.8\%\\ \; & = & 29\% \end{array}$$ [Eqn 7]

$$\begin{array}{lll} \text{Specificity} & = & \frac{TN}{TN+FP}\times 100\\ \; & = & \frac{41}{46}\times 100\\ \; & = & 89.1\%\\ \; & = & 89\% \end{array}$$ [Eqn 8]

$$\begin{array}{lll} \text{Positive predictive value} & = & \frac{TP}{TP+FP}\times 100\\ \; & = & \frac{15}{20}\times 100\\ \; & = & 75\% \end{array}$$ [Eqn 9]

$$\begin{array}{lll} \text{Negative predictive value} & = & \frac{TN}{TN+FN}\times 100\\ \; & = & \frac{41}{78}\times 100\\ \; & = & 52.6\%\\ \; & = & 53\% \end{array}$$ [Eqn 10]

$$\begin{array}{lll} \text{False - positive rate} & = & \frac{FP}{FP+TN}\times 100\\ \; & = & \frac{5}{46}\times 100\\ \; & = & 10.86\%\\ \; & = & 10.9\% \end{array}$$ [Eqn 11]

$$\begin{array}{lll} \text{False - negative rate} & = & \frac{FN}{TP+FN}\times 100\\ \; & = & \frac{37}{52}\times 100\\ \; & = & 71.15\%\\ \; & = & 71.2\% \end{array}$$ [Eqn 12]

$$\begin{array}{lll} \text{Accuracy - efficiency of the test} & = & \frac{TP+TN}{TP+FN+FP+TN}\times 100\\ \; & = & \frac{15+41\times 100}{98}\\ \; & = & \frac{56}{98}\times 100\\ \; & = & 57.1\% \end{array}$$ [Eqn 13]

$$\begin{array}{lll} \text{Prevalence of malaria (using microscopy)} & = & \frac{TP+FN}{TP+FP+FN+TN}\times 100\\ \; & = & \frac{52}{98}\times 100\\ \; & = & 53.1\%\\ \; & = & 53\% \end{array}$$ [Eqn 14]

**TABLE 7:** Diagnostic accuracy of malaria rapid diagnostic test using microscopy as the gold standard.

mRDT Result (Index Test Result)	Microscopy result (Reference Standard)		Total
	Positive	Negative	
Positive	15	5	20
	(TP)	(FP)	(TP + FP)
Negative	37	41	78
	(FN)	(TN)	(FN + TN)
**Total**	**52**	**46**	**98**
	**(TP + FN)**	**(FP + TN)**	**(TP + FN + FP + TN)**

FN, false negative; FP, false positive; mRDT, malaria rapid diagnostic test; TN, true negative; TP, true positive.

## Discussion

This study aimed at comparing the diagnostic accuracy of mRDT with microscopy amongst under five children so as to deploy mRDT for prompt diagnosis and treatment of malaria amongst children presenting to our hospital.

### Malaria prevalence

The prevalence of malaria in this study using microscopy as a reference diagnostic test was 53% (95% CI = 46% – 60%). This value was lower than that recorded by Samadoulougou et al.^4^ in Burkina Faso, who had a prevalence of 65.0% amongst the under five children during the rainy season, but higher than that of Oyeyemi et al.^5^ in a descriptive, cross-sectional study in Ijebu Ode, Western Nigeria, who had a prevalence of 36.8% for mRDT in their study population. The differences in the prevalence results may be because of differences in the endemicity of malaria from the different malaria epidemiological zones where the studies were carried out.

### Parasite species distribution

The species of malaria parasite identified in all study participants was *P. falciparum.* This agrees with a similar study carried out by Oyeyemi et al.^5^ in South-Western part of Nigeria comparing microscopy and rapid diagnostic test as malaria diagnostic tools where only *P. falciparum* species of the malaria parasites was identified in all the study participants. However, Agomo et al.^6^ in Lagos, Nigeria, found that *P. falciparum* was seen in 95.2% of the cases as either mixed infection with *P. malariae* (3.6%) or as a mono infection (91.6%). These results agree with literature findings that *P. falciparum* is responsible for more than 95% of malaria infections in the tropics.^4,7,8^ Therefore HRP2-based mRDTs are more economical and the preferred options for parasitological diagnosis of malaria than the enzyme-based mRDTs in the tropics. In addition, HRP2-based rapid diagnostic tests (HRP2-mRDTs) can withstand the heat and temperature fluctuations of tropical Africa better than the enzyme-based RDTs, where refrigeration and air conditioning are not always feasible.

### Distribution of parasite count

The result revealed that 52 children (53.1% [95% CI = 40.7% – 61.3%]) out of the 98 were positive for the microscopy test. Thirty seven (71.2%) out of the 52 children who were positive for the microscopy were found to be negative with mRDT (false negative). This gave a high false negative mRDT test when compared with the result of the microscopy in this study. This is very significant in this study, as this may have contributed to the low sensitivity reported. Sensitivity is the proportion of people with disease (malaria) who will have a positive result when tested with the diagnostic test kit (mRDT in this case) in the diagnosis of malaria.

The microscopy further showed that the parasite count of (+) made up 67% of the total population of patients with positive microscopy. Using this ‘plus’ system scale of scoring to calculate the parasite density, it therefore means that about 67% of the participants who had positive results with microscopy had a parasite density of between 10 and 90 parasites/µL of blood.^9^ This is below the parasite density threshold (100 parasites/µL which is equivalent to 0.002% parasitaemia) that can be reliably detected by mRDT for malaria diagnosis.^10^ The low yield of positive results with the mRDT in this study agrees with the fact that the malaria rapid test result positivity is low at low parasite density.^10^ Amadi et al.^11^ in Port Harcourt, South – South Nigeria, found that mRDT sensitivity was only 45% when the parasite density was below 100 parasite/µL. These results show that rapid test would not give justifiable results as most of the low parasite density cases could escape detection.

### Comparing the performance of malaria rapid diagnostic test and microscopy

#### Determination of sensitivity and specificity of malaria rapid diagnostic test

The sensitivity and specificity of the mRDT in this study were 29% and 89%, respectively. This means that the mRDT kit (CareStat^TM^) used in this study will be capable of detecting correctly (giving a positive result) only 29 out of 100 children with malaria infection and will give a negative result in 89 out of 100 patients without malaria infection. The very low sensitivity recorded in this study as against the WHO recommendations of about 95% may be because of a high false negative rate of 71.2% (37/52 × 100) of mRDT as compared to the microscopy. The high false negative rate is similar to the findings of Berhane et al. where only 10 out of the 50 microscopically confirmed *P. falciparum* infected specimens were confirmed positive (i.e. 40 mRDT negative results out of 50 confirmed specimens microscopically), with all the lots of mRDTs used in the study giving an 80% false negativity proportion.^12^ The possible explanation for these findings may include a low parasite density below the threshold of mRDT positivity (< 100 asexual parasites/µL or < 0.002% of red blood cells infected).^10,13^ Other studies have also shown some degree of false negative result for mRDT because of hyperparasitaemia, deletion or mutation of *HRP-2* gene and the prozone effect (which is defined as false-negative or falsely low results in immunological reactions because of excess of either antigens or antibodies). This will eventually affect the sensitivity of the test.^13,14,15^

The low sensitivity of this study is in agreement with the research conducted by Oyeyemi et al. in Ijebu Ode, western part of Nigeria, who reported a sensitivity and specificity of 42.5% and 87.1%, respectively.^5^ Garba et al.^16^ at Gusau, Nigeria, who worked on comparison of microscopy and rapid diagnostic test in under five children, got a sensitivity of 9.1%. However, the sensitivity of 29% in this study is far below that obtained by researchers like Ezeudu at the children’s out-patient clinic and children’s emergency room of Nnamdi Azikiwe University Teaching Hospital (NAUTH) Nnewi, Nigeria, who reported a sensitivity and specificity of 80% and 93.8%, respectively.^17^ Xiaodong et al.^18^ in China also found that the CareStat rapid diagnostic test had a sensitivity of 89.68% and a specificity of 98.26% compared to the gold standard microscopy method for the detection of malaria. Variations in sensitivity between the different studies may be attributed to differences in the types of RDTs used or test methodology and skills of the microscopist. The implication of the low sensitivity in this study is that in areas with low malaria parasitaemia, a negative result should be cross-checked with a microscopy and clinical acumen of the physician to rule out possibilities of false negative results with the mRDT. However, a high specificity of 89% in this study implied that mRDT may be used in primary healthcare centres by community health workers to rule out the absence of malaria where microscopes are hardly seen or where the required human expertise is lacking.

It was also noted from this study that five (10.9%) of 46 children whose microscopy results were negative were positive with the rapid test (false positive). This may be as a result of persistent antigen of the malaria parasite in the blood even after parasite clearance following adequate anti-malaria treatment of the index cases. The persistent antigenaemia may have contributed to the high specificity recorded in this study. This agreed with the work of Batwala et al.^19^ in rural health centres in Uganda that compared the accuracy of rapid diagnostic tests and microscopy where the overall specificity of Paracheck (a form of HRP2-based mRDTs) was lower than that of the microscopy.

The percentage agreement of positive results of mRDT and parasite count using microscopy was the highest (100%) at parasite count of (++++) and the lowest (14.3%) with parasite count of (+). Many of these (+) using microscopy were missed by the rapid test, thereby giving a low yield in the positivity of the mRDT and, consequently, the sensitivity of the malaria kit at this level of parasite count. This result agreed with the work of Sani et al. in Sokoto, Northern Nigeria, where it was found that the sensitivity of RDT increased consistently from 33% at low parasite density to 93% at high parasite density.^20^ The explanation for this may either be as a result of reduced sensitivity of the mRDT at low parasite count as documented by these authors or the over-diagnosis of malaria by the laboratory scientists at low parasite density.^20,21,22^ Kahama-Maro et al.^7^ in Dar es Salaam found that only 2.1% of the 178 slides that were reported positive by health facility routine microscopy were actually positive by expert microscopy.^22^ This low percentage (2.1%) may be as a result the possible lack of the rigorous and diligent commitment required to report parasite count of (+) (i.e. 0–10 parasites in 100 high power field) on the part of most microscopists, because of the pressure of work they have to cope with whilst carrying out their routine work. Some of this parasite count of (+) may also be because of artefact resulting from either poor blood film preparation or using reagents with sediments in staining the blood smears.

The specificity of this study was comparable with most of other researches. However, the low sensitivity of this study agreed with the work carried out by Kahama-Maro et al. in Dar es Salaam, who found a low sensitivity. The low sensitivity in this study may not be completely explained only by the parasite density of the malaria as documented by researchers like Mawili-Mboumba.^23^

#### Positive and negative predictive values of malaria rapid diagnostic test

The positive and negative predictive values in this study were 75% and 53%, respectively. This result is slightly different from the findings of Falade et al.,^7^ who had a positive and negative predictive values of 65.6% and 86.1%, respectively. The positive predictive value of 75% means that the kit has the capability of confirming malaria with a precision of 75%, whilst the negative predictive value of 53% means that the mRDT is good in ruling out malaria, thus giving the clinician the confidence that a negative test excluded malaria in about 53% of cases.

The false positive and negative rates in this study were 10.9% and 71.2%, respectively. This false negative rate is quite high. Several factors may account for this high rate, which may include low parasite density. According to WHO, false-negative results can be caused by any or a combination of the following:

- the procurement and use of poor-quality RDTs
- use of the wrong comparator for the diagnostic test, such as poor-quality microscopy for cross-checking negative RDT
- poor transport and storage conditions for RDTs, with sustained exposure to high temperature
- operator errors during performance and/or interpretation of RDT results (more rarely)
- deletion or mutation of *HRP-2* gene.

#### Association between parasite density and parasite count using microscopy

The linear regression plot (Figure 3) showed that the log parasite density increased by a unit value as the parasite count increased by 1.2 (*p* < 0.001). This relationship also indirectly affects the positivity of mRDT because the higher the parasite count, the greater the percentage agreement of positive results of mRDT with microscopy. It therefore means that the parasite density is directly related to the mRDT positivity.

### Limitation

There was only one microscopist who regularly performed malaria microscopy for clinical care of patients in the study location where the volume of work could sometimes be high. There was no cross-checking of a predetermined percentage of the slides by a second microscopist.

## Conclusion

The sensitivity and specificity of mRDT compared with microscopy diagnosis of malaria in this study were found to be 29% and 89%, respectively. There was a significant correlation between parasite count and parasite density (*p* < 0.001). We therefore advocate a more sensitive kit that can detect the malaria parasite at low density for future use, especially to improve the sensitivity of the mRDT kits in malaria management, where trained microscopists for malaria diagnosis are not available.
